# Acute postoperative complications after scleral fixation of Akreos AO60 intraocular lens

**DOI:** 10.1093/jscr/rjag159

**Published:** 2026-03-16

**Authors:** Piergiacomo Grassi, Panagiotis Tsoutsanis, Robert Henderson

**Affiliations:** Department of Ophthalmology, Northern Care Alliance NHS Foundation Trust, Rochdale Infirmary, Whitehall St, Rochdale OL12 0NB, United Kingdom; School of Medical Sciences, Faculty of Biology, Medicine and Healthy, The University of Manchester, Stopford Building, Oxford Road, Manchester, M13 9PL, United Kingdom; Department of Ophthalmology, Northern Care Alliance NHS Foundation Trust, Rochdale Infirmary, Whitehall St, Rochdale OL12 0NB, United Kingdom; School of Medical Sciences, Faculty of Biology, Medicine and Healthy, The University of Manchester, Stopford Building, Oxford Road, Manchester, M13 9PL, United Kingdom; Reuben College, University of Oxford, Parks Rd, Oxford OX1 3QP, United Kingdom; Moorfields Eye Hospital NHS Foundation Trust, 162 City Rd, London EC1V 2PD, United Kingdom

**Keywords:** Akreos, case report, endophthalmitis, scleral fixation, toxic anterior segment syndrome

## Abstract

We report three cases of postoperative exogenous endophthalmitis and toxic anterior segment syndrome (TASS) following vitrectomy for dislocated intraocular or crystalline lenses and scleral fixation of an Akreos intraocular lens (IOL) with Gore-Tex sutures. To our knowledge, postoperative exogenous endophthalmitis and TASS have not been previously described with this technique. A 67-, 56-, and 36-year-old underwent pars plana vitrectomy, lens removal or vitreolensectomy, and Akreos IOL scleral fixation. Three days later, cases 1 and 3 required vitreous biopsy and intravitreal Vancomycin/Amikacin for culture-positive endophthalmitis (*Staphylococcus epidermidis* and *Streptococcus pneumoniae*). Case 2 underwent removal of a dislocated fluocinolone implant, biopsy, and intravitreal antibiotics; negative cultures supported a diagnosis of TASS. Scleral-fixated IOLs carry risks of endophthalmitis and TASS. Large corneal wounds increase risk; meticulous wound construction is essential. Akreos IOLs do not prevent anterior migration of steroid implants. Immediate management is crucial.

## Introduction

Scleral-fixation (SF) of posterior chamber intraocular lenses (PC-IOLs) may be preferred when a PC-IOL is anatomically desired, such as in patients with corneal decompensation, or when iris fixation or anterior chamber-IOL (AC-IOL) are not possible; or when concurrent pars plana vitrectomy (PPV) is required to address retained lens material, dislocated IOL, or subluxed crystalline lens. Excellent visual outcomes have been reported with Akreos AO60 IOLs, with significant improvements in best-corrected visual acuity (BCVA) [1], validating this technique and becoming a popular alternative to iris-fixated IOLs, AC-IOLs or SF of PC-IOLs. We report 3 eventful cases after PPV and ab-externo scleral-fixation (AESF) of Akreos AO60 IOL with Gore-Tex sutures (GTS) in absence of preoperative risk factors (RFs).

## Cases presentation

**Figure 1:**
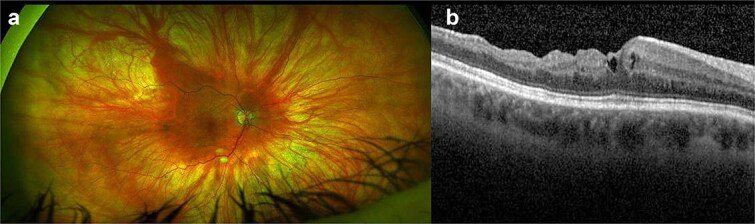
(a, b) Right eye coloured fundus photograph and macular OCT horizontal scan after right vitreous biopsy and intravitreal injection of antibiotics showing residual epiretinal membrane and loss of retinal lamination.

**Case 1:** A 67-year-old myope lady attended for right dislocated IOL and epiretinal membrane (ERM). Her pre-operative right BCVA measured 20/60, examination revealed a monopiece IOL/bag complex subluxed temporally and symptomatic ERM. She underwent 25G PPV, IOL/bag complex removal through a 6-mm clear cornea incision (CCI), ERM peeling and fixation of Akreos IOL with GTS. CCI and conjunctiva were carefully sutured. Two days later she presented with ocular pain; right BCVA measuring hand motion, exam showed fibrin in anterior chamber (AC). She underwent right AC washout, vitreous biopsy and intravitreal injection (IVI) of Vancomycin/Amikacin 0.1 ml. She was started on intensive oral/topical antibiotics and steroids, followed 2 days later by right IVI of Ceftazidime 0.1 ml. The vitreous cultures were positive for *Staphylococcus Epidermidis*. Three months later her right BCVA had improved to 20/40, macular OCT showed residual ERM and loss of retinal lamination (Fig. 1a and b).

**Figure 2:**
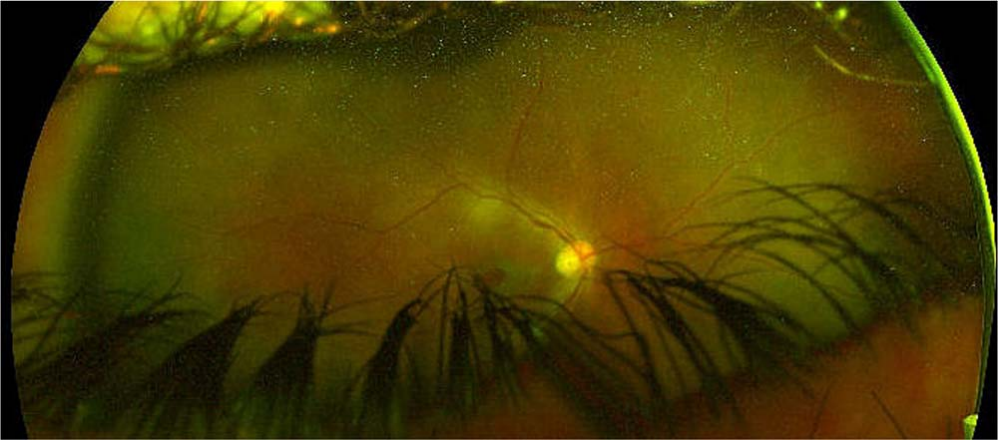
Right eye coloured fundus photograph after anterior chamber washout, 23G vitrectomy, vitreous cavity washout, large pseudomembrane peeling from posterior pole and a second intravitreal injection of vancomycin/amikacin 0.1 ml + periocular injection of triamcinolone showing a large full thickness macular hole.

**Case 2:** A 36-year-old male attended for right subluxation of crystalline lens. The initial right BCVA measured 20/60, examination revealed partially subluxed crystalline lens in the vitreous cavity. He underwent 23G vitreolensectomy with fixation of Akreos IOL with GTS through a 3.4 mm CCI; all 23G sclerotomies and CCI were sutured. Twenty days later his right BCVA had improved to 20/17. Four weeks postoperatively, he presented with right intraocular inflammation and a loose corneal suture which was removed with sterile technique. The following day, he presented with ocular pain, light perception (LP) acuity, and 2-mm hypopyon. He underwent vitreous biopsy and IVI of Vancomycin/Amikacin 0.1 ml, was started on intensive oral/topical antibiotics and steroids. Two days later exam showed right siedel positive CCI and a choroidal effusion. Vitreous cultures were positive for *Streptococcus Pneumoniae* and he underwent AC washout + corneal resuturing. Ten days later, the patient represented with ocular pain, and LP acuity. He had an 8-mm hypopyon and dense pupillary membrane. He underwent further AC washout, 23G vitrectomy, vitreous cavity washout and a large pseudomembrane peeled from posterior pole. A second IVI of Vancomycin/Amikacin 0.1 ml + periocular injection of triamcinolone were performed. Two weeks postoperatively, the pain resolved, his BCVA measured counting fingers (CF), exam showed clear media, flat retina and full thickness macular hole, the second vitreous cultures being negative (Fig. 2).

**Figure 3:**
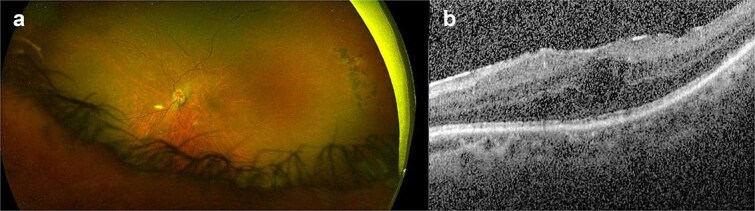
(a, b) Left eye coloured fundus photograph and macular OCT horizontal scan after left vitreous biopsy, intravitreal injection of antibiotics and removal of the dislocated steroid implant from the anterior chamber showing residual epiretinal membrane and cystoid macular oedema.

**Case 3:** A 56-year-old male with sarcoid panuveitis, left filtering Baerveldt tube for uveitic glaucoma attended for left dislocated IOL. The patient had previously undergone left dexamethasone and fluocinolone acetonide intravitreal implants for cystoid macular oedema (CMO) and was on oral mycophenolate mofetil. BCVA measured 20/60, examination revealed nasal half of monopiece IOL/bag complex dislocated in AC and resolved CMO. He underwent uneventful 27G vitrectomy, removal of IOL/bag complex and AESF of Akreos IOL with GTS through a 4.8 mm CCI which was sutured. Three days later, he presented with left BCVA of CF and discomfort. Exam showed corneal oedema and 1-mm hypopyon. He underwent vitreous biopsy and IVI of Vancomycin/Amikacin 0.1 ml and was started on intensive oral/topical antibiotics and steroids for suspected endophthalmitis. Four days later vitreous cultures and gram stain were negative, exam showed the flucinolone acetonide implant dislocated in AC and pseudohypopyon. He underwent AC washout and removal of the dislocated implant. 1 month later, BCVA increased to 20/60; exam showed clear media, ERM and CMO (Fig. 3a and b), toxic anterior segment syndrome (TASS) was confirmed. Three months later BCVA and CMO remained unchanged.

## Discussion

Post-operative hypotony, ocular hypertension, hyphema, vitreous haemorrhage, choroidal detachment, CMO, corneal oedema, IOL opacification and purulent scleritis from conjunctival erosion from GTS have been reported after Akreos AO60 AESF [1–4]. To our knowledge post-operative exogenous endophthalmitis (POEE – early or delayed) and TASS have not been previously reported. Case 1 and 2 highlight the potential risk of a large CCI for the introduction of an Akreos IOL with threaded GTS through its eyelets. The potential for ingress of micro-organisms through large, sutured wounds remains. In elderly patients, CCIs take longer to repair and premature sutures removal should be avoided. Careful wound construction and accurate suturing remain vital. Minimizing CCI size may reduce the risk, though folding the lens with GTS in place may be challenging. Also, resting a PC-IOL on the surgical drape whilst threading the GTS prior to introduction into the eye is a further RF for POEE. Importantly both POEE cases were recorded during the COVID-19 pandemic, when patients underwent surgery wearing surgical face masks and turnover times were longer between cases [5]. Case 3 shows that not all eyes with uveitic glaucoma can undergo topical hypotensive medications washout as previously suggested [6], and that an AESF Akreos IOL is not an effective barrier in vitrectomized eyes to intravitreal steroid implants migration into AC. Interestingly, none of our cases presented with conjunctival erosion/GTS exposure, which are recognized RFs for POEE. PPV is an established treatment for eyes with rhegmatogenous retinal detachment (including challenging cases complicated by proliferative vitreoretinopathy) and with vitreous haemorrhage [7–13], but less so for different conditions [14–16]. Although an excellent option for the management of surgical aphakia in absence of capsular support, surgeons should be well-aware of risks for POEE, TASS and anterior migration of intravitreal steroid implants. In case of post-operative pain and blurred vision, strong suspicion for POEE should arise, even if presentation is delayed. Immediate management with vitreous biopsy, intravitreal antibiotics injection and oral/topical steroids are required.

## Data Availability

The datasets used and/or analyzed during the current study are available from the corresponding author on reasonable request.
